# 

**Published:** 1836

**Authors:** 


### INDEX.

| | PAGE |
|---|---|
| A | |
| Abortion, manual assistance in | 110 |
| Age of Medical Men | 319 |
| Agents, Mechanical | 225 |
| Chemical | 255 |
| Vital | 255 |
| Amenorrheoa cured by Turpentine | 506 |
| Antispasmodics | 439 |
| Aneurismal tumors obliterated | 324 |
| Aneurism of Thoracic Aorta | 344 |
| Annelida | 611 |
| Apoplexy, pathology of | 473 |
| Diagnosis of | 480 |
| Treatment of | 486 |
| Circumstances predisposing to | 479 |
| Arteries, vital properties of | 467 |
| In Erectile tissue | 325 |
| Ascitis cured by Injections | 276 |
| Astringents | 443 |
| In Cholera | 21 |
| Autumnal Diseases in Mississippi | 161 |
| B | |
| Blood | 541 |
| changed by repeated bleedings | 653 |
| Disease of | 278 |
| Lesions of | 67 |
| Pleuritic coat of | ib. |
| Blood-letting | 262 |
| in Cholera | 19 |
| in Scarlet Fever | 647 |
| Blue Sulphur Springs | 159 |
| Brain, injury of | 237 |
| Breath | 539 |
| Breschet's operation for Hydrocele | 507 |
| C | |
| Camphorated ointment in Hydrocele | 329 |
| Catalogue of Ohio plants | 567 |
| Cataract cured in old age | 369 |
| Cathartics in Cholera | 20 |
| Catechu in mercurial disease | 329 |
| Charcoal in Lungs | 298 |
| Chalybeates in Dropsy | 158 |
| Cholera, anatomical characters of | 81 |
| Fantasies | 13 |
| Resuscitation of | 24 |

| | PAGE |
|---|---|
| Colchicum in cure of acute Rheumatism | 333 |
| Chyle | 536 |
| Cincinnati College | 524 |
| Manifesto | 193 |
| Circulation of Blood in Arteries and veins | 472 |
| vegetables | 418 |
| Club Foot | 399 |
| Consumptives Retreat | 161 |
| Contagion and Miasm, organic | 401 |
| Contractions of Urethra | 464 |
| Cough, Remarks on | 666 |
| Credulity | 270 |
| Croup cured by Sulp. Cupri | 653 |
| Crustacea | 612 |
| D | |
| Death of Leaves by Lead fumes | 157 |
| Delirium Tremens | 678 |
| Dew point, method of finding | 162 |
| Diabetes Melites cured by Cantharides | 331 |
| Diet | 520 |
| Digitalis, a specific for Delirium Tremens | 678 |
| Endermic, use of in Dropsy | 131 |
| Disease, self-limited | 285 |
| Diuretic Medication | 461 |
| Drake's letter to Committee | 171 |
| Doctor Julius of Prussia | 369 |
| Dropsy, cases of | 131 |
| Dura mater, Inflammation of | 315 |
| E | |
| Editorial Note | 550 |
| Remarks | 550 |
| Editorial and publishing arrangements | 154 |
| Editor's proposed route of travel | 680 |
| Elementary Textures of Vegetables | 601 |
| Emetics in Cholera | 19 |
| Endogenous growth | 603 |
| Epilepsy cured by Indigo | 506 |
| Epidemic Cholera, nitrate of silver in | 109 |
| Erysipelas, observations on | 124 |
| Ergot in Pulmonary Hæmorrhage | 678 |

PAGE

Estep's case of Fistula 377
Excitants 257
Expérimental Enquiry into nervous system 469
Exogenous plants 604

F

False Testimony 272
Final causes of organic action 600
Fistula of Urethra by Dr. Estep 379
Fluids of Animals modified by food 542
Food and Flesh of Animals, Relations of 535
Fracture of Radius 135
In child, accident mistaken ib.
Friends of Medical Reform, Address to 197

G

Gangrene of the Lungs following Small-Pox 692
General and Pathological Anatomy, study of 368
Geological survey 376
Getter, Mrs. Murdered 28
Greenville Epsom Springs 159

H

Hernia Strangulated 53
Radical Cure of 575
Home, Sir Everard's Piracy 353
Horner on Asiatic Cholera 81
Hot Douching in Sprains and Bruises 163
Hossack, David M. D. Obituary, 513
Human Combustion 78
Hydrophobia, case of D. Wonderly 592
Symptoms of 593
Postmortem appearances 594
Hypertrophy, muscular coat of stomach 298
Hysteria, Dr. Woodward on 41

I

Ice in Cholera 20
Inflammation of Spinal Dura mater, 137
Infusoria 608
Intermittent Fever in the West 372
Iodine 257
Itch, Remedy for 324

J

Jaundice, Cure for 324
Journal of Pharmacy 618

K

Kreosote, Facts relating to 464
Cure for Diabetes Melitus 676

L

Language, Ambiguity of 274
Lead, sheet in dressing wounds 289
Lepra Vulgaris 324
Treatment of 120

PAGE

Lesions of the Blood 57
of sensation and volition 389
Ligature for Brachial Aneurism 331
Liquor Amni, absorption of 366
Lithotomy 164
of Dr. McDowell 225
and Wounded Rectum 216
Little's Case of Siamese connexion 39

M

Maddox's Annomalous Disease 389
Malignant Pustule 662
Mammary Abcess, treatment of 122
Manual Strangulation 25
Experiments 30
Maryland Academy of Science 519
Materia Medica 248
by A. G. Thompson 426
Mechanical Functions of organization 601
Medical Cyclopædia 444
College of Ohio Reform 169
College of Louisiana 608
Lexington 610
Department of Cincinnati College 685
Experience 266
History of the West 679
Observations 375
Schools 154, 607
Society of Tennessee 77
Memoir Dupuytren 146
Memorial of Physicians of Ohio 170
Mercury 258
Mercurials in Cholera 20
Mercurial Transudation 163
Metastasis of disease from Bowels to Brain 691
Meteorology 547
Meteorological Notices by D. D. Owen 550
Miasmata in Air, and detection of 133
Miasm and Contagion 401, 526
Milk 537
secreted from Scrotum 327
sickness caused by Rhus Radicans 243
Minerals and Mineral waters Analyzed 37
Mnemonic Nomenclature 687
Mode of preserving Animal Bodies 673
Mollusca 610
Monstrosities 414
Morphia 429
Mount's Case of Strangulated Hernia 53
M'Guire's Case of Gun Shot Wound 382

N

Narcotics 426
in Cholera 20

PAGE
Necrosed Sequestrum Separated 223
Nervous Symptoms Illustrated 299
System and Irritability of Muscles 465
Nitrate of Silver in Cholera 109
in Tonsilitis 157
Nux Vomica 232
O
Operation on Tumor of Face 206
Ophthalma, treatment of 117, 122
Ophthalmia, Catarrhal 114
Catarrho—Rheumatic 116
Rheumatic 114
Os Femoris, fracture of 509
Our Periodical 521
Overton's Case of human Combustion 78
Oxygen Gas in Cholera 21
P
Pain and Constitutional Effects from Couching 368
Paralysis from disease of arterial System 490, 647
the Result of Apoplexy 488
Diagnosis of 492
Treatment of 500
Paraphlegia 494
Perspiration 539
Phrenology, Benefits of 156
Physiology of Respiration 361
By Jun. Editor 599
Physicians wanted at Sandwich Islands 165
Pneumogastric Nerve, researches on 129
Polypifera, 608
Post mortem examination in Cholera 15
Professor Jameson 371
McDowell 526
Prolapsus Ani 510
Purgatives in Typhus 294
Pulmonary Consumption 451
R
Recto Vaginal, communication 328
Refrigerants 265
Report of Committee on Med. College of Ohio 172
Report on Meteorology 552
Researches on Blood 656, 659
Rheumatic Ophthalmia 114
S
Saline Draughts 20
Scrofula 106
Secretion of Vegetables 622
Sedatives 260
Siamese connection discovered 38

PAGE
Sick Stomach 243
Silk oiled, in warm-water Dressing 328
Solidism and Humoralism 18
Sponges, propagation of 607
Statistics, Austrian 223
Stimulants in Cholera 20
Sulphate of Copper in Croup 652
Superstition 269
Syphilis cured without mercury 139
T
Tannin, action of on Salafiable bases 464
Tartrate Antimony, in large doses 130
Temperature of Air 552
Theory, False 274
Thermometer for 1828 551
Thyroid Gland Extirpated 338
Tic Doloroux 676
Tonics 440
Treatment of Syphilis without mercury 139
Tumors of the Face 205
Turpentine Solidified by Magnesia 675
U
Unskilful Surgery 202
Urine 539
Urinary calculus Lithotripsy 511
V
Vapor Baths in ocular affections 462
Vegetable Nutrition 616
Venous Injection in Cholera 20
Vessels of Plants 602
Veratrum Viride 335
Vertebrata 612
W
Western Academy of Natural Sciences 155
Western Medical Schools 607
Willoughby University 369
Winter Retreat for Consumptives 161
Woodward on Hysteria 41
Word from the Proprietors 532
Worms, Intestinal 307, 301
perforating Intestines 305
Pathology of 305
Symtoms of 308
Causes of 311
Treatment of 312
Wounds by Air and Steam Guns 102
Treatise on, by Dupuytren 97
of the heart, by Dr. Simmons 382
by incision and puncture 107
by gun powder 163
Z
Zoophytes 606

